# Persistent Borafluorene Radicals

**DOI:** 10.1002/anie.201909627

**Published:** 2020-02-03

**Authors:** Wenlong Yang, Kelsie E. Krantz, Lucas A. Freeman, Diane A. Dickie, Andrew Molino, Gernot Frenking, Sudip Pan, David J. D. Wilson, Robert J. Gilliard

**Affiliations:** ^1^ Department of Chemistry University of Virginia 409 McCormick Rd./ PO Box 400319 Charlottesville VA 22904 USA; ^2^ Department of Chemistry and Physics La Trobe Institute for Molecular Science La Trobe University Melbourne 3086 Victoria Australia; ^3^ Fachbereich Chemie Philipps-Universität Marburg Hans-Meerwein-Straße 35043 Marburg Germany

**Keywords:** borafluorenes, boron, carbenes, radicals

## Abstract

N‐Heterocyclic carbene (NHC)‐ and cyclic (alkyl)(amino)carbene (CAAC)‐stabilized borafluorene radicals have been isolated and characterized by elemental analysis, single‐crystal X‐ray diffraction, UV/Vis absorption, cyclic voltammetry (CV), electron paramagnetic resonance (EPR) spectroscopy, and theoretical studies. Both the CAAC–borafluorene radical (**2**) and the NHC–borafluorene radical (**4**) have a considerable amount of spin density localized on the boron atoms (0.322 for **2** and 0.369 for **4**). In compound **2**, the unpaired electron is also partly delocalized over the CAAC ligand ^carbene^C and N atoms. However, the unpaired electron in compound **4** mainly resides throughout the borafluorene π‐system, with significantly less delocalization over the NHC ligand. These results highlight the Lewis base dependent electrostructural tuning of materials‐relevant radicals. Notably, this is the first report of crystalline borafluorene radicals, and these species exhibit remarkable solid‐state and solution stability.

## Graphical abstract

**Crystalline, isolable** borafluorene radicals that are stabilized by N‐heterocyclic carbene or cyclic (alkyl)(amino)carbene ligands have been synthesized. They exhibit remarkable solution and solid‐state stability and were characterized by elemental analysis, single‐crystal X‐ray diffraction, UV/Vis absorption, cyclic voltammetry, EPR spectroscopy, and theoretical studies.

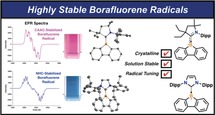

Borafluorene,^1^ a five‐membered aromatic heterocycle, has been studied extensively as a building block for molecular materials, and new chemistries utilizing this platform are rapidly emerging.^2^ Indeed, the incorporation of main group heteroatoms into the π‐system of polycyclic aromatic hydrocarbons has led to functional materials with wide‐ranging applications in organic light‐emitting diodes, organic thin film transistors, and photovoltaic devices.^3^ The neutral tricoordinate borafluorene (Figure 1 a) has been explored extensively and shows interesting reactivity with alkynes,2b, 4 azides,2c, 5 1,2‐dipolar substrates,^6^ and carbenes.^7^ In addition, borafluorene‐based polymers exhibit high luminescence quantum yields.2f While the structure of the first borafluorene cation (i.e., borafluorenium) was reported by Nöth over three decades ago,1b we recently described the synthesis, optical tuning, and thermochromism of borafluorenium species supported by carbenes (Figure 1 b).^8^ Anionic tricoordinate borafluorene was targeted by Rivard and co‐workers by chemical reduction strategies; however, hydrogen atom abstraction (from THF or residual water) led to the formation of the tetracoordinate hydridoboron adduct (Figure 1 c).^9^ It is noteworthy that the intermediate radical species of borafluorene has remained elusive.

**Figure 1:**
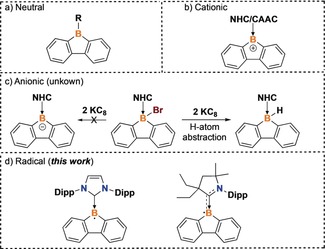
Structural and electronic diversity in the tricoordinate borafluorene framework: a) neutral, b) cationic, c) anionic, and d) radical.

Radicals possess energetically accessible electrons, and therefore the materials community has targeted stable organic radicals for their potential in charge transport and storage applications, and they have played a key role as active layer materials in devices.^10^ Indeed, the unpaired electron can facilitate access to uncommon doublet spin states, which are advantageous with regard to the function of optoelectronic materials.^11^ Moreover, redox‐active radicals are beneficial as they may perform in an ambipolar fashion, being susceptible to both oxidation or reduction processes.^12^ However, many radicals are short‐lived, and thus the development of stable materials‐relevant radical compounds that are well‐poised for synthetic elaboration still presents a major challenge for the scientific community. Boron‐centered radicals have been studied extensively in recent years because of their importance in both pure and applied chemistry.^13^ Neutral boryl radicals germane to this report include Lewis base stabilized BH_2_^.^,^14^ BR^1^R^2.^,^15^ and borole radicals.^16^ In order to expand the radical‐based chemistry to molecular platforms that are stable and relevant to materials, we report the synthesis, molecular structures, UV/Vis absorption, electrochemistry, electron paramagnetic resonance (EPR) spectroscopy, and theoretical studies of N‐heterocyclic carbene (NHC)‐ and cyclic (alkyl)(amino)carbene (CAAC)‐stabilized borafluorene radicals (**2** and **4**; Figure 1 d). Notably, compounds **2** and **4** represent the first examples of isolated borafluorene radicals. These deeply colored radical species are remarkable in the sense that they are persistent in solution for at least three months and are indefinitely stable in the solid state under inert atmosphere.

The stabilizing ability of the strongly σ‐donating and π‐accepting CAAC ligand is now well‐established.^17^ We reduced the 9‐bromo‐9‐borafluorene–CAAC adduct **1** 8 with one equivalent of potassium graphite (KC_8_) in toluene at room temperature, which produced the CAAC‐stabilized borafluorene radical **2** as a purple crystalline solid in 69 % isolated yield (Scheme 1 a). Based on the electronic properties of carbenes, we expected that the synthesis of an NHC‐stabilized borafluorene radical would lead to a species with dramatically different chemical and physical properties. Therefore, the NHC‐stabilized borafluorene radical **4** was isolated in 47 % yield as a blue crystalline solid by reduction of the 9‐bromo‐9‐borafluorene–NHC adduct **3** 8, 9 with KC_8_ under similar conditions (Scheme 1 b). It is noteworthy that both radical compounds are remarkably stable in the solid state, retaining their color and crystallinity under inert atmosphere indefinitely. Surprisingly, solution‐phase stability tests demonstrated that **2** and **4** are also stable in dry toluene at room temperature for over three months.

**Scheme 1:**
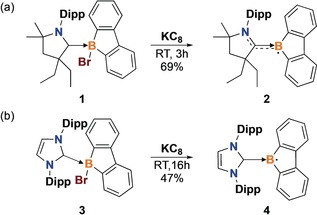
Synthesis of borafluorene radicals. Dipp=2,6‐diisopropylphenyl.

Air‐ and moisture‐sensitive purple (**2**) and blue (**4**) single crystals suitable for X‐ray crystallographic analysis were obtained for both radicals (Figure 2, see Table S1 in the Supporting Information for additional crystallographic information).^18^ The tricoordinate boron center exhibits trigonal planar geometry in both structures. The interplanar angles between the central carbene ring and the borafluorene units are 21.42(9)° for **2** and 33.15(9)° for **4**, which differ from the calculated interplanar angles of model compounds where N‐Dipp is replaced with N‐Me (31.941° for **2^NMe^** and 45.006° for **4^NMe^**). The carbene–boron (B1−C1) bonds are similar in **2** [1.551(2) Å] and **4** [1.550(2) Å] and are comparable to those in cationic borafluorene compounds.^8^ However, the C−B bonds in the borole ring of **4** [1.554(2) and 1.553(2) Å] are shorter than those in **2** [1.611(2) and 1.605(2) Å], and agree with the calculated values (1.572 and 1.570 Å in **2^NMe^** and 1.549 and 1.549 Å in **4^NMe^**). This observation is consistent with the radical being more localized over the borafluorene π‐system in the NHC‐stabilized compound **4**. In compound **2,** the radical is more delocalized onto the carbene ligand, and this result clearly highlights the enhanced π‐acceptor property of the CAAC.

**Figure 2:**
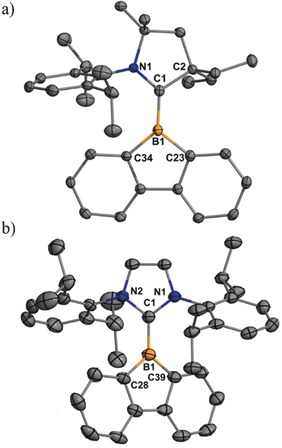
Molecular structures of **2** (a) and **4** (b). Thermal ellipsoids set at 50 % probability; H atoms and one of the two molecules in the asymmetric unit of **4** were omitted for clarity. Selected experimental [calculated] bond lengths [Å] and angles [°]: **2**: B1–C1 1.551(2) [1.530], B1–C23 1.611(2) [1.572], B1–C34 1.605(2) [1.570]; C1‐B1‐C34 131.89(14) [125.29], C1‐B1‐C23 125.96(14) [131.00], C34‐B1‐C23 101.84(13) [103.44]; **4**: B1–C1 1.550(2) [1.548], B1–C28 1.554(2) [1.549], B1–C39 1.553(2) [1.549]; C1‐B1‐C39 128.20(15) [127.66], C1‐B1‐C28 127.03(15) [127.66], C39‐B1‐C28 104.77(14) [104.67]. Calculated values are for model compounds **2^NMe^** and **4^NMe^**.

The UV/Vis absorption spectra of **2** and **4** in hexane are shown in Figure 3 a. Both radicals exhibit strong absorption in the visible region. It is noteworthy that **2** (*λ*_max_=550 nm) showed a 60 nm blue‐shift compared to **4** (*λ*_max_=610 nm). Theoretical calculations (B3LYP‐D3(BJ)/def2‐TZVP) were carried out with model compounds **2^NMe^** and **4^NMe^**, which provided geometries consistent with the X‐ray structures (Figure 2; see the Supporting Information for computational details). TD‐DFT (ωB97XD/def2‐SVP) calculations of UV/Vis absorbance gave a 70 nm blue‐shift in **2^NMe^** compared to **4^NMe^**, consistent with the 60 nm blue‐shift in the experimental spectra. Broad absorption bands were calculated at 496 nm for **2^NMe^** and 553 nm for **4^NM^** ^e^, and were characterized as SOMO→LUMO transitions (π→π*), where the SOMO is the singly occupied MO. Shorter wavelength absorptions at about 245 nm were characterized as SOMO→LUMO+1 for **2^NMe^**, and SOMO→LUMO+2 for **4^NMe^**. Plots of the SOMO and LUMO of **2^NMe^** and **4^NMe^** are shown in Figure 3 b.

**Figure 3:**
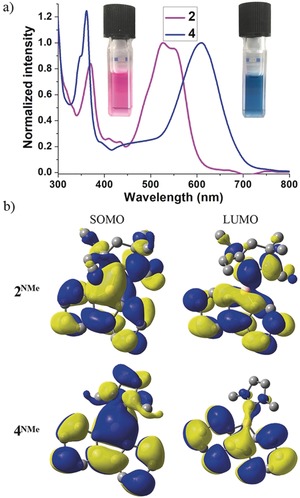
a) UV/Vis absorbance spectra of **2** and **4** in hexane. b) Plots of SOMO and LUMO for **2^NMe^** and **4^NMe^**.

EPR spectroscopy unambiguously confirmed the radical nature of compounds **2** and **4**, which display drastically different multiple‐line spectra in toluene at room temperature centered at *g=*1.9971 and 2.0085, respectively (Figure 4 a). Despite the complexity of the hyperfine splitting, simulated spectra were obtained for both radicals (see Figures S1 and S2 for plots and Table S2 for hyperfine coupling parameters). These data clearly indicate that the chemical environment of the unpaired electron in **2** is substantially different from that in **4**. The shape of the SOMO reveals that the unpaired electron in both **2^NMe^** and **4^NMe^** is significantly delocalized over both the borafluorene and the CAAC or NHC moieties (Figure 3 b). The calculations of spin density (plotted in Figure 4 and tabulated in Tables S3–S5) show that the net spin density located on the CAAC (0.38 e^−^) is larger than that on the NHC (0.16 e^−^), which is in line with the strong π‐acidity of the former ligand compared to the latter.

**Figure 4:**
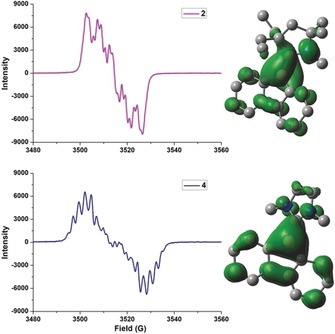
Experimental EPR spectra of **2** and **4** in toluene solution at 298 K (left). Spin density contour plots of **2^NMe^** and **4^NMe^** (right).

Energy decomposition analysis in combination with natural orbital for chemical valence (EDA‐NOCV) theory^19^ was performed to shed additional light on the nature of the bonding in compounds **2^NMe^** and **4^NMe^**. The numerical results of EDA‐NOCV using singlet L=CAAC^Me^ or NHC^Me^ and doublet borafluorene as interacting fragments are provided in Table S6. The consideration of the alternative choice, doublet [L]^−^ and singlet [borafluorene]^+^ as interacting fragments, shows that the former scheme is better suited to describe the bonding in these compounds as it gives a lower orbital value, Δ*E*_orb_, than the latter (see Table S7). The data in Table S6 reveal that the intrinsic interaction between L and borafluorene is rather strong, being −135.1 (**2^NMe^**) and −117.9 kcal mol^−1^ (**4^NMe^**). The contributions from the covalent (Δ*E*_orb_) and Coulomb attraction (Δ*E*_elstat_) towards the intrinsic attraction are similar, with the former being slightly more dominant over the latter in **2^NMe^** and a reverse situation in **4^NMe^**. The dispersion is only responsible for 3–4 % of the total attraction. The breakdown of Δ*E*_orb_ into pairwise orbital interactions shows that the strongest orbital interaction, Δ*E*_orb(1)_, originates from the [L]→[borafluorene] σ‐donation (67–72 % of total Δ*E*_orb_), whereas the next important orbital term, Δ*E*_orb(2)_, is due to the [L]←[borafluorene] π‐back‐donation, which correlates with the SOMO. The associated charge flow (red→blue) is nicely reflected in the corresponding plots of deformation densities (Δ*ρ*) along with the charge eigenvalues in Figure 5. The stronger σ‐donating and π‐accepting ability of the CAAC compared to the NHC is clearly understood from the relative size of Δ*E*_orb(1)_ and Δ*E*_orb(2)_, and from the corresponding charge eigenvalues, |*ν*|.

**Figure 5:**
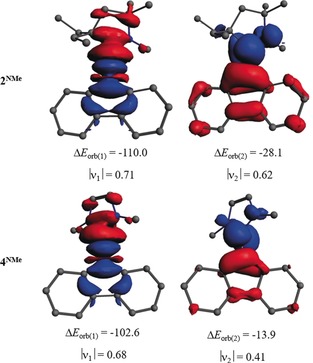
Shapes of the deformation densities Δ*ρ*_(1)_ and Δ*ρ*_(2)_, which are associated with the orbital interactions Δ*E*_orb(1)_ and Δ*E*_orb(2)_ in **2^NMe^** and **4^NMe^**, and eigenvalues |*ν_n_*| of the charge flow. The isosurface value is 0.001. The color code of the charge flow is red→blue.

To gain insight into the redox properties of **2** and **4**, cyclic voltammograms (CV) were recorded in THF (Figure 6). Although the anions were chemically unstable under specified conditions (see Figure 1 c),^9^ the anionic species are stable on an electrochemical timescale, and were observed by the reversible reduction waves at *E*_1/2_=−1.82 and −2.25 V for **2** and **4**, respectively (referenced against the ferrocene/ferrocenium (Fc/Fc^+^) redox couple, see Figures S3 and S4 for multiple scans). Compound **2** has a lower reduction potential than **4**, which can be attributed to the greater π‐accepting ability of the CAAC ligand. Both compounds show an irreversible oxidation, which is due to THF coordination to the cationic species, a behavior that we recently explored in detail.^8^

**Figure 6:**
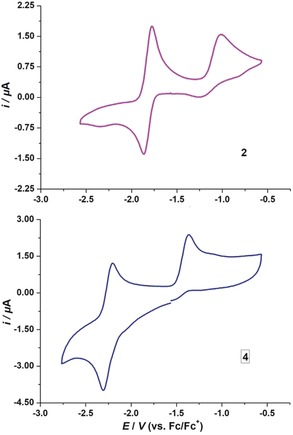
Cyclic voltammograms of **2** and **4** in THF/0.1 m [*n*Bu_4_N][PF_6_] at room temperature. Scan rate: 100 mV s^−1^.

In conclusion, we have synthesized and fully characterized the first isolated examples of borafluorene radicals. The optical and electrochemical properties were tuned by varying the carbene ligand. Compared to the CAAC‐stabilized borofluorene radical, the NHC‐stabilized radical exhibited a red‐shifted absorption and a higher reduction potential in CV experiments. EPR and DFT analyses clearly showed that both radicals possess a significant amount of spin density on boron (0.322 for **2** and 0.369 for **4**). In the CAAC‐stabilized borafluorene radical, the unpaired electron is delocalized over the carbene ligand carbon and nitrogen atoms. In contrast, the unpaired electron is mainly located within the borafluorene π‐system in the NHC‐stabilized radical. Unlike the majority of heterocyclic organic radicals, the borafluorene radicals reported herein are remarkably stable in the solid state indefinitely, and in dry toluene for at least three months. As the borafluorene framework is now ubiquitous in many areas of materials chemistry, we expect that these results will pave a way for new borafluorene‐based redox‐active materials. Efforts are now underway to extend the radical chemistry to multi‐boron‐doped materials with extended conjugation.

## Conflict of interest

The authors declare no conflict of interest.

## Supporting information

As a service to our authors and readers, this journal provides supporting information supplied by the authors. Such materials are peer reviewed and may be re‐organized for online delivery, but are not copy‐edited or typeset. Technical support issues arising from supporting information (other than missing files) should be addressed to the authors.

Supplementary
