# Supplementary material for: Persistent Borafluorene Radicals
Source: Angew Chem Int Ed Engl. 2020 Feb 3;59(10):3850–4. doi: 10.1002/anie.201909627 (PMC7064902; doi:10.1002/anie.201909627)
Supplement: Supplementary file 1 — Supplementary [file ANIE-59-3850-s001.pdf]

Angewandte Chemie
Eine Zeitschrift der Gesellschaft Deutscher Chemiker

Supporting Information

Persistent Borafluorene Radicals

Wenlong Yang, Kelsie E. Krantz, Lucas A. Freeman, Diane A. Dickie, Andrew Molino, Gernot Frenking, Sudip Pan, David J. D. Wilson,* and Robert J. Gilliard, Jr.**

anie_201909627_sm_miscellaneous_information.pdf

Table of Contents

General Procedures 3

Experimental Procedures 3

X-ray data collection details 4

Theoretical Calculations 6

References 18

SUPPORTING INFORMATION

General Procedures: All manipulations were carried out under an atmosphere of purified argon using standard Schlenk techniques or in an MBRAUN LABmaster glovebox equipped with a –37 °C freezer. All reaction solvents were distilled over sodium/benzophenone. Deuterated solvents were purchased from Acros Organics and Cambridge and distilled over sodium/benzophenone prior to use. Glassware was oven-dried at 190 °C overnight. Single crystal X-ray diffraction data were collected on a Bruker Kappa APEXII Duo system equipped with a fine-focus sealed tube (Mo Kα, λ = 0.71073 Å). The structures were solved and refined using the Bruker SHELXTL Software Package 2 within OLEX2.3. Elemental analyses were performed at Midwest Microlab, 7212 N. Shadeland Ave., Suite 110, Indianapolis, IN 46250. UV-Vis absorption spectra were recorded using a Cary 60 UV-Vis Spectrophotometer. EPR spectra were recorded using a Bruker X-Band EMX spectrometer (Bruker Biospin, Billerica, MA) equipped with an ER 4123D dielectric resonator. All EPR spectra were recorded using a 100 G magnetic field sweep, 1 G modulation, and 2.0-milliwatt incident microwave power at a temperature of 298 K. IPr[1] and CAAC[2] were prepared according to published procedures. Compounds **1** and **3** were synthesized according to literature procedures.[3]

Experimental Procedures

Synthesis of compound 2:

To a toluene (15 mL) solution of **1** (55 mg, 0.10 mmol), KC_8 (14 mg, 0.10 mmol) was added slowly at room temperature. The solution turned to purple immediately. After stirring for 3 hours, the mixture was filtered then toluene was removed under vacuum. The obtained solid was extracted with hexanes (20 mL) then concentrated to 5 mL. After recrystallization at –37 °C, 33 mg of purple solid was obtained (69%). Anal. Calcd for $C_{34}H_{43}BN$: C, 85.70; H, 9.10; N, 2.94 %. Found: C, 85.06; H, 8.94; N, 3.00 %.

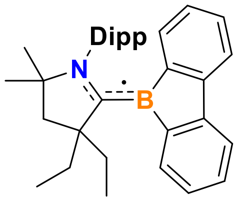

Synthesis of compound 4:

To a toluene (20 mL) solution of **3** (31 mg, 0.05 mmol), KC_8 (7 mg, 0.05 mmol) was added slowly at room temperature. The solution turned to blue slowly. After stirring for 16 hours, the mixture was filtered then toluene was removed under vacuum. The obtained solid was extracted with hexanes (30 mL) then concentrated to 10 mL. After recrystallization at –37 °C, 13 mg of blue solid was obtained (47%). Anal. Calcd for $C_{39}H_{44}BN_2$: C, 84.92; H, 8.04; N, 5.08 %. Found: C, 84.65; H, 8.33; N, 5.17 %.

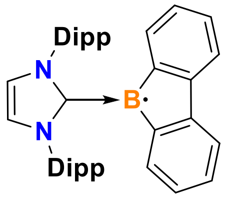

X-ray data collection details

A single crystal of **2** or **4** was coated with Paratone oil and mounted on a MiTeGen MicroLoop. The X-ray intensity data were measured on a Bruker Kappa APEXII Duo system equipped with a fine-focus sealed tube (Mo K_α, λ = 0.71073 Å) and a graphite monochromator. The frames were integrated with the Bruker SAINT software package[4] using a narrow-frame algorithm. Data were corrected for absorption effects using the multi-scan method (SADABS).[4] The structures were solved and refined using the Bruker SHELXTL Software Package[5] within APEX3[4] and OLEX2.[6] Non-hydrogen atoms were refined anisotropically. Hydrogen atoms were placed in geometrically calculated positions with U_{iso} = 1.2U_{equiv} of the parent atom (U_{iso} = 1.5U_{equiv} for methyl). In **4**, one toluene solvent molecule was found to be disordered over three positions. The relative occupancies were freely refined with the restriction that their sum equal one. The anisotropic displacement parameters and the ring geometry of the disordered atoms were constrained. A second toluene solvent could not be adequately modeled with or without restraints. Thus, the structure factors were modified using the PLATON SQUEEZE[6] technique, in order to produce a "solvate-free" structure factor set. PLATON reported a total electron density of 50 e^- and total solvent accessible volume of 275 $Å^3$.

Table S1. Crystallographic data for **2** and **4**

	**2**	**4**
Chemical formula	$C_{34}H_{43}BN$	$C_{42.47}H_{47.97}BN_2$
Formula weight (g/mol)	476.50	597.28
Temperature (K)	100(2)	100(2)
Crystal size (mm)	0.038 x 0.237 x 0.246	0.421 x 0.452 x 0.594
Crystal habit	red block	dark blue block
Crystal system	monoclinic	triclinic
Space group	P 2_1/n	P -1
a	13.0506(17)	13.9246(8)
b	16.253(2)	15.7107(9)
c	13.1446(15)	19.5015(11)
α (°)	90	102.444(2)
β (°)	93.534(4)	107.343(2)
γ (°)	90	105.778(2)
Volume ($Å^3$)	2782.8(6)	3709.0(4)
Z	4	4
Density (calculated) (g/cm^3)	1.137	1.070
μ (mm^{-1})	0.064	0.061
Theta range (°)	2.00 to 27.53	1.42 to 27.94
Index ranges	-16<=h<=16,-21<=k<=21, -17<=l<=17	-18<=h<=17, -19<=k<=20, -25<=l<=25
Reflections collected	31048	72769
Independent reflections	6404 [R(int) = 0.0751]	17780 [R(int) = 0.0345]
Data / restraints / parameters	6404 / 0 / 333	17780 / 1 / 839
Goodness-of-fit on F^2	1.007	1.029
R_1 [I>2σ(I)]	0.0481	0.0505
wR_2 [all data]	0.1159	0.1431

Theoretical Calculations

The geometry optimizations and the subsequent harmonic frequency calculations for the model **2**$^{\mathbf{NMe}}$ and **4**$^{\mathbf{NMe}}$ compounds were carried out at the B3LYP-D3(BJ)[7]/def2-TZVP[8] level inclusive of solvation (Truhlar's SMD model, dichloromethane solvent) using the Gaussian 16 suit of program.[9] TD-DFT calculations employed ωB97XD[10]/def2-SVP[8] (solvation with SMD,[11] dichloromethane), with 20 states modelled. EPR parameters were computed at the B3LYP/EPR-II level[12a] in ORCA 4.2.0 program[12b] to obtain the hyperfine coupling values. The EPR spectra are simulated in the EasySpin program.[13]

The energy decomposition analysis (EDA) in combination with natural orbital for chemical valence (NOCV) method was performed at the B3LYP-D3(BJ)/TZ2P+//B3LYP-D3(BJ)/def2-TZVP level using the ADF (2017.101) program package.[14]

In the EDA method, the interaction energy (ΔE_{int}) between two prepared fragments is divided into three energy terms, viz., the electrostatic interaction energy (ΔE_{elstat}), which represents the quasiclassical electrostatic interaction between the unperturbed charge distributions of the prepared atoms, the Pauli repulsion (ΔE_{Pauli}), which is the energy change associated with the transformation from the superposition of the unperturbed electron densities of the isolated fragments to the wavefunction that properly obeys the Pauli principle through explicit antisymmetrization and renormalization of the product wavefunction, and the orbital interaction energy (ΔE_{orb}), which is originated from the mixing of orbitals, charge transfer and polarization between the isolated fragments. Lastly, since we have used dispersion corrected functional, it adds the dispersion contribution to the interaction energy.

Therefore, the interaction energy (ΔE_{int}) between two fragments can be defined as:

$$\Delta E_{int} = \Delta E_{elstat} + \Delta E_{Pauli} + \Delta E_{orb} + \Delta E_{disp} \qquad (1)$$

The EDA-NOCV calculation combines charge and energy decomposition schemes to divide the deformation density, $\Delta\rho(\mathbf{r})$, associated with the bond formation into different components (σ, π, δ) of a chemical bond. From the mathematical point of view, each NOCV, ψ_i is defined as an eigenvector of the deformation density matrix in the basis of fragment orbitals.

$$\Delta P\psi_i = v_i\psi_I \qquad (2)$$

In EDA-NOCV, ΔE_{orb} is given by the following equation

$$\Delta E_{orb} = \sum_k \Delta E_k^{orb} = \sum_{k=1}^{N/2} \nu_k [-F_{-k}^{TS} + F_k^{TS}] \qquad (3)$$

where, $-F_{-k}^{TS}$ and F_k^{TS} are diagonal Kohn-Sham matrix elements corresponding to NOCVs with the eigenvalues $-v_k$ and v_k, respectively. The ΔE_k^{orb} terms are assigned to a particular type of bond by visual inspection of the shape of the deformation density, $\Delta\rho_k$. The EDA-NOCV scheme thus provides both qualitative ($\Delta\rho_{orb}$) and quantitative (ΔE_{orb}) information about the strength of orbital interactions in chemical bonds. More details about EDA-NOCV and its application can be found in recent reviews.[15]

SUPPORTING INFORMATION

Cartesian coordinates computed at the RO-B3LYP-D3(BJ)/def2-TZVP (SMD, dichloromethane) level of theory.

2^{NMe}

Ee = -974.025634211

B	-0.54693400	-0.04769800	-0.24800600
N	1.73655800	-1.07882200	-0.66835800
C	0.98200500	-0.07731400	-0.21249500
C	1.89905400	0.96319200	0.41919500
C	3.18496000	0.15890500	0.70735700
H	4.07955900	0.75404200	0.53543500
H	3.20340800	-0.16076400	1.74767100
C	3.15416500	-1.07765100	-0.20789900
C	4.12811600	-0.97262500	-1.38411700
H	4.10302100	-1.86519400	-2.00994400
H	5.14206500	-0.87120000	-0.99548400
H	3.91390000	-0.10518300	-2.00806500
C	3.42939800	-2.36143600	0.57732700
H	2.70443800	-2.48182600	1.38375000
H	4.42709300	-2.30816000	1.01688700
H	3.39298600	-3.24378400	-0.06325100
C	1.32621500	1.60572100	1.69557600
H	2.11296800	2.22038400	2.13907400
H	0.52260200	2.28906100	1.41838900
C	0.80412200	0.63307100	2.74897100
H	1.57071100	-0.07325000	3.07444200
H	-0.04292800	0.05524100	2.37609200
H	0.46855800	1.18051500	3.63253700
C	2.17429100	2.03964000	-0.66945800
H	2.68168500	1.55386200	-1.50429400
H	1.22453100	2.39313300	-1.06687800
C	2.99961000	3.23770300	-0.20863900
H	3.94780300	2.93361600	0.24001000
H	2.46317900	3.84488600	0.52137400
H	3.23105200	3.87861600	-1.06235700
C	-1.55388100	1.15760200	-0.31227600
C	-1.43923200	2.54445900	-0.45230100
H	-0.46797700	3.01570200	-0.49877200
C	-2.56763100	3.35912100	-0.52523100
H	-2.45002400	4.43127800	-0.62812400
C	-3.84510200	2.80545900	-0.46633600
H	-4.71733500	3.44483100	-0.52629500
C	-3.99876000	1.42787800	-0.32698400
H	-4.99164900	0.99621100	-0.27719800
C	-2.87262700	0.61816000	-0.24909600
C	-2.82908000	-0.83841000	-0.07293200
C	-3.89272300	-1.70942200	0.12850300
H	-4.91383300	-1.34614500	0.12179100
C	-3.63632400	-3.05954000	0.36094200
H	-4.45844500	-3.74640200	0.52072800
C	-2.32176700	-3.52293000	0.40951700
H	-2.12825000	-4.56909000	0.61480800
C	-1.25735000	-2.64839700	0.20095300
H	-0.24861600	-3.03464200	0.26438400
C	-1.48270000	-1.29581700	-0.07049200
C	1.31271600	-2.06386400	-1.64516100
H	1.38422900	-3.07694800	-1.25062200
H	0.28854400	-1.86586900	-1.93961000
H	1.95063800	-1.99371300	-2.52749300

4^{NMe}

Ee = -792.196643564

N	-2.65179000	0.85226500	0.66299700
N	-2.65172600	-0.85237900	-0.66296300
C	-1.82324500	-0.00004600	0.00004700
C	-3.96943100	0.53454600	0.41500300
H	-4.78057400	1.08648700	0.85490100
C	-3.96939200	-0.53479500	-0.41492700
H	-4.78048500	-1.08688700	-0.85472500
C	0.67126700	-1.21663400	0.15227700
C	0.48604500	-2.58737400	0.38029900
H	-0.51446000	-2.99821100	0.45302700
C	1.57575500	-3.43992800	0.52321800
H	1.41503900	-4.49776700	0.69564300
C	2.88099200	-2.94228200	0.45048800
H	3.72255900	-3.61483700	0.56324600
C	3.10042200	-1.58182700	0.24317700
H	4.11390000	-1.19861200	0.20070000
C	2.01501900	-0.72500500	0.09956500
C	2.01498300	0.72508700	-0.09942000
C	3.10035700	1.58194000	-0.24307000
H	4.11384600	1.19876400	-0.20054200
C	2.88087600	2.94236800	-0.45051200
H	3.72241500	3.61495400	-0.56329900
C	1.57561900	3.43994300	-0.52334900
H	1.41485800	4.49775700	-0.69588600
C	0.48593900	2.58735100	-0.38039800
H	-0.51457500	2.99815200	-0.45320700
C	0.67120600	1.21664600	-0.15221900
B	-0.27518100	-0.00001700	0.00009900
C	-2.23415300	-1.90059800	-1.58091800
H	-1.21450600	-1.70503600	-1.89969900
H	-2.28083500	-2.87562300	-1.09767700
H	-2.89405500	-1.89478300	-2.44659400
C	-2.23434700	1.90070700	1.58074500
H	-1.21484600	1.70508500	1.89996100
H	-2.28070100	2.87559000	1.09719200
H	-2.89454600	1.89526200	2.44619400

Table S2. The computed g and the employed hyperfine fine coupling constants (in MHz) of different atomic centers in **2** and **4** for the simulations.

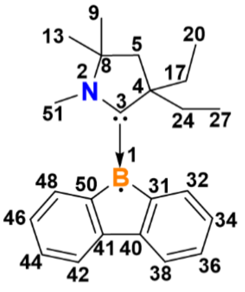 2^{NMe}	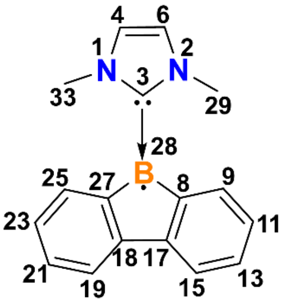 4^{NMe}

2	Centers	g_{iso}	4	Centers	g_{iso}
		2.0026			2.0026
		A_{iso}			A_{iso}
	B(1)	8.20		N(1)	7.45
	N(2)	4.94		N(2)	7.45
	C(3)	-2.10		B(28)	20.15
	C(40), C(41)	1.86		C(3)	-23.03
	C(32), C(48)	1.86		C(6)	-2.05
	C(36), C(44)	1.86		C(4)	-2.05
	C(50), C(31)	1.86		C(27)	-20.63
				C(25)	7.22
				C(23)	-7.60
				C(21)	9.37
				C(19)	-8.74
				C(18)	5.67
				C(17)	5.67
				C(15)	-8.74
				C(13)	9.37
				C(11)	-7.60
				C(9)	7.21
				C(8)	-20.63
				C(33)	1.28
				C(29)	1.27
				H attached to C(13)	-7.89
				H attached to C(9)	-6.23
				H attached to C(25)	-6.23
				H attached to C(21)	-7.89

Table S3. Spin density comparison of borafluorene.

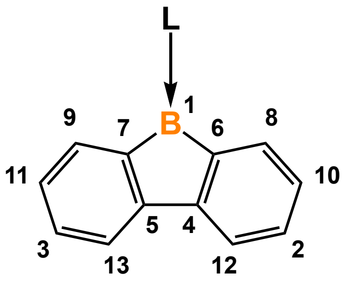

Heterocycle Spin Density Analysis			
**Atom ID**	**4^{Me} (NHC)**	**2^{Me} (CAAC)**	**Δ(4-2)**
**B(1)**	0.369	0.322	0.047
**C(2)**	0.061	0.037	0.023
**C(3)**	0.061	0.039	0.022
**C(4)**	0.055	0.033	0.022
**C(5)**	0.055	0.034	0.020
**C(6)**	0.045	0.023	0.022
**C(7)**	0.045	0.026	0.019
**C(8)**	0.044	0.030	0.014
**C(9)**	0.044	0.033	0.011
**C(10)**	0.014	0.007	0.006
**C(11)**	0.014	0.006	0.007
**C(12)**	0.006	0.005	0.002
**C(13)**	0.006	0.004	0.002

Table S4. Spin density of **2^NMe^**.

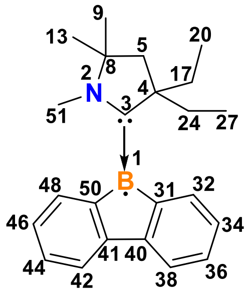

Atom I.D.	Spin Density
B(1)	0.322
C(3)	0.223
N(2)	0.122
C(44)	0.039
C(36)	0.037
C(41)	0.034
C(48)	0.033
C(40)	0.033
C(32)	0.030
C(50)	0.026
C(31)	0.023
C(17)	0.009
C(24)	0.008
C(34)	0.007
C(46)	0.006
C(8)	0.005
C(13)	0.005
C(38)	0.005
C(42)	0.004
C(9)	0.004
C(27)	0.003
C(51)	0.002
C(5)	0.001
C(20)	-0.001
C(4)	-0.001

Table S5. Spin density of **4^NMe^**.

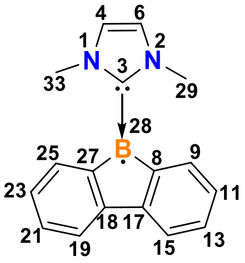

Atom I.D.	Spin Density
B(28)	0.369
C(3)	0.077
C(13)	0.061
C(21)	0.061
C(17)	0.055
C(18)	0.055
C(8)	0.045
C(27)	0.045
C(9)	0.044
C(25)	0.044
N(2)	0.026
N(1)	0.026
C(6)	0.017
C(4)	0.017
C(11)	0.014
C(23)	0.014
C(15)	0.006
C(19)	0.006
C(29)	0.000
C(33)	0.000

Table S6. EDA-NOCV results of 2NMe and 4NMe compounds at the B3LYP-D3(BJ)/TZ2P+//B3LYP-D3(BJ)/def2-TZVP level. The interacting fragments are singlet L = [CAACMe] or [NHCMe] and doublet [borafluorene]. Energy values are given in kcal/mol.

Energy terms	Orbital interactions	2^{NMe} [CAAC^{Me}] + [borafluorene]	4^{NMe} [NHC^{Me}] + [borafluorene]
ΔE_{int}		-135.1	-117.9
ΔE_{Pauli}		200.3	180.2
ΔE_{disp}[a]		-12.4 (3.7%)	-8.4 (2.8%)
ΔE_{elstat}[a]		-158.7 (47.3%)	-147.7 (49.5%)
ΔE_{orb}[a]		-164.4 (49.0%)	-142.0 (47.6%)
$\Delta E_{orb(1)}$[b]	[L]→[borafluorene] σ donation	-110.0 (66.9%)	-102.6 (72.3%)
$\Delta E_{orb(2)}$[b]	[L]←[borafluorene] π backdonation	-28.1 (17.1%)	-13.9 (9.8%)
$\Delta E_{orb(rest)}$		-26.3 (16.0%)	-25.5 (18.0%)

[a] The values in parentheses give the percentage contribution to the total attractive interactions ΔE_{elstat}+ ΔE_{orb} + ΔE_{disp}. [b] The values in parentheses give the percentage contribution to the total orbital interactions $\Delta E_{orb.}$

Table S7. EDA-NOCV results of $\mathbf{2^{NMe}}$ and $\mathbf{4^{NMe}}$ compounds at the B3LYP-D3(BJ)/TZ2P+//B3LYP-D3(BJ)/def2-TZVP level. The interacting fragments are doublet L^- = [CAAC^{Me}]$^-$ or [NHC^{Me}]$^-$ and singlet [borafluorene]$^+$. Energy values are given in kcal/mol.

Energy terms	Orbital interactions	2^{NMe} [CAAC^{Me}]$^-$ + [borafluorene]$^+$	4^{NMe} [NHC^{Me}]$^-$ + [borafluorene]$^+$
ΔE_{int}		-305.6	-310.5
ΔE_{Pauli}		219.4	204.7
ΔE_{disp}[a]		-12.4 (2.4%)	-8.5 (1.6%)
ΔE_{elstat}[a]		-242.7 (46.2%)	-250.4 (48.6%)
ΔE_{orb}[a]		-270.0 (51.4%)	-256.4 (49.8%)
$\Delta E_{orb(1)}$[b]	[L]$^-$→[borafluorene]$^+$ σ donation	-129.7 (48.0%)	-122.3 (47.7%)
$\Delta E_{orb(2)}$[b]	[L]$^-$→[borafluorene]$^+$ π donation	-97.7 (36.2%)	-90.7 (35.4%)
$\Delta E_{orb(rest)}$		-42.6 (15.8%)	-43.4 (16.9%)

[a]The values in parentheses give the percentage contribution to the total attractive interactions ΔE_{elstat}+ ΔE_{orb} + ΔE_{disp}.

[b]The values in parentheses give the percentage contribution to the total orbital interactions ΔE_{orb}.

Table S8. Experimental EPR spectra parameter list for **2** and **4**.

Parameter	**2**	**4**
Receiver Gain	1.00e+0.004	1.00e+0.004
Phase	0	0
Harmonic	1	1
Mod. Frequency	100.000 kHz	100.000 kHz
Mod. Amplitude	1.000 G	1.000 G
Center Field	3515.000 G	3515.000 G
Sweep Width	100.000 G	150.000 G
Resolution	2048 POINT	2048 POINT
Sweep Time	20.972 s	20.972 s
Microwave Frequency	9.824000 GHz	9.873000 GHz
Microwave Power	1.992e+000 mW	2.007e+000 mW

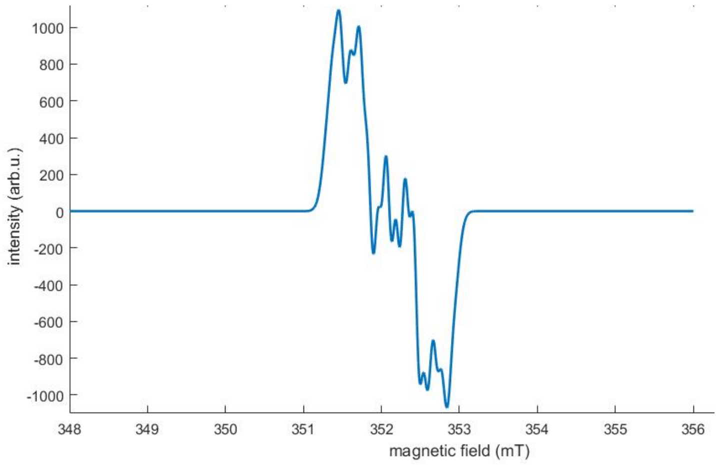

Figure S1. Simulated EPR spectrum considering 11 different nuclei having large spin density (B1, N2, C3, C41, C40, C32, C36, C44, C48, C50, C31) of **2**.

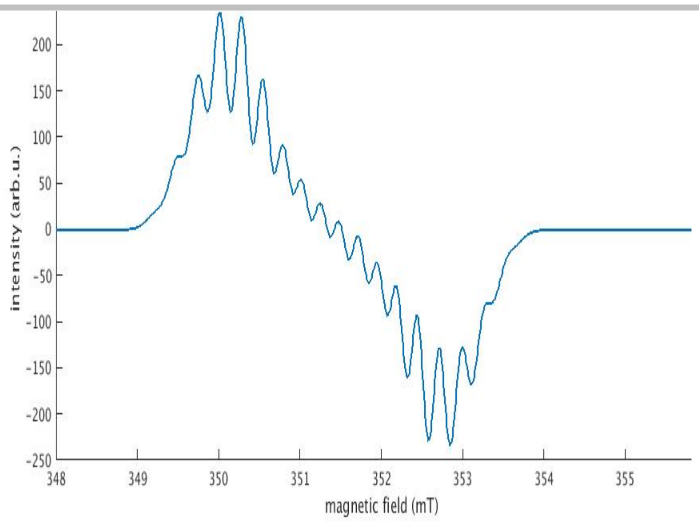

Figure S2. Simulated EPR spectrum of **4**.

EPR details in EasySpin

For **2**

```
Sys=orca2easyspin('Path \bfluorene_caac_epr_b3lyp_epr2_aiso-adip-selected-total-molecule-revised-small-number.prop')
Sys =   struct with fields:
      S: 0.5000
      g: 1.9970
   Nucs: '11B,14N,13C,13C,13C,13C,13C'
      A: [8.2000 4.9400 -2.1000 1.8600 1.8600 1.8600 1.8600]
      n: [1 1 1 2 2 2 2]
   lwpp: 0.1000
Range: [348 356]
   mwFreq: 9.8420
Temperature = 298
garlic(Sys,Exp)
```

For **4**

```
Sys=orca2easyspin('Path\bfluorene_nhc_epr_b3lyp_epr2_aiso-adip-selected-H.prop')
Sys =   struct with fields:
        S: 0.5000
      xyz: [36×3 double]
   Charge: 0
        g: [2.0023 2.0026 2.0028]
   gFrame: [-1.5710 1.7872 3.1415]
     Nucs: 'N,N,C,C,H,C,H,C,C,H,C,C,H,C,C,C,C,C,H,C,C,H,C,B,C,C'
        A: [26×3 double]
   AFrame: [26×3 double]
Exp.mwFreq = 9.85
Exp.Range = [348 356]
Sys.lwpp = [0.23]          % Gaussian broadening
Temperature = 298
garlic(Sys,Exp)
```

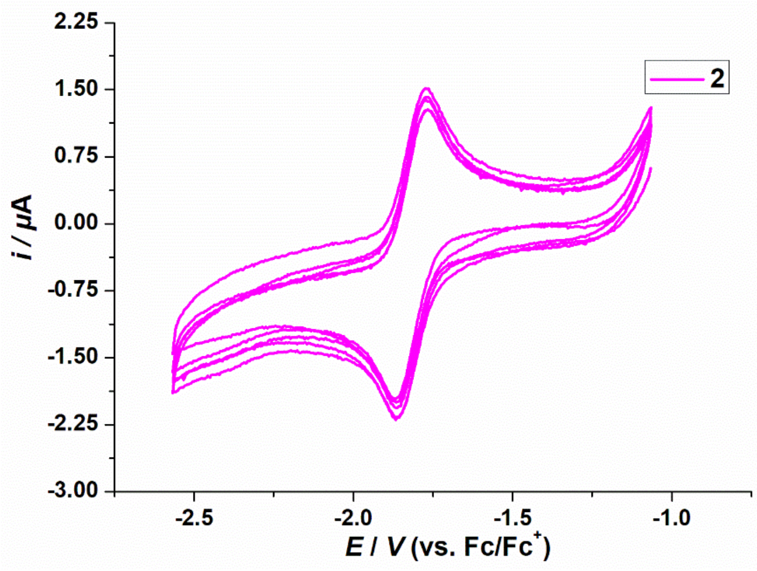

Figure S3. Cyclic voltammogram of **2** in THF/0.1M [*n*Bu_4N][PF_6] at room temperature. Scan rate: 100 mVs^{-1}, 5 scans.

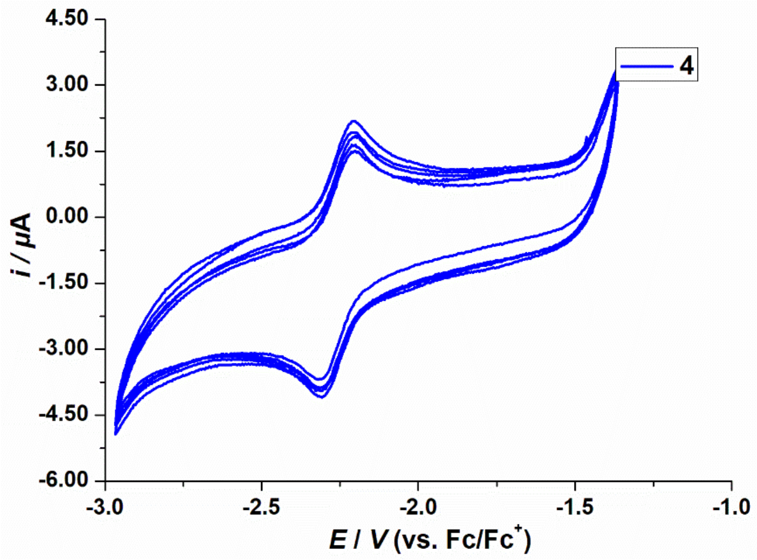

Figure S4. Cyclic voltammogram of **4** in THF/0.1M [nBu_4N][PF_6] at room temperature. Scan rate: 100 mVs^{-1}, 5 scans.

References

[1] A. J. Arduengo, R. Krafczyk, R. Schmutzler, H. A. Craig, J. R. Goerlich, W. J. Marshall, M. Unverzagt, *Tetrahedron* **1999**, *55*, 14523-14534.

[2] R. Jazzar, R. D. Dewhurst, J.-B. Bourg, B. Donnadieu, Y. Canac, G. Bertrand, *Angew. Chem. Int. Ed.* **2007**, *46*, 2899-2902.

[3] W. Yang, K. Krantz, F. Lucas, D. Diane, A. Molino, A. Kaur, D. Wilson, R. J. Gilliard, *Chem. Eur. J.*, *0*.

[4] R. Stegmann, G. Frenking, *Journal of Computational Chemistry* **1996**, *17*, 781-789.

[5] G. Sheldrick, *Acta Crystallogr. A* **2015**, *71*, 3-8.

[6] O. V. Dolomanov, L. J. Bourhis, R. J. Gildea, J. A. K. Howard, H. Puschmann, *J. Appl. Crystallogr.* **2009**, *42*, 339-341.

[7] (a) A. D. Becke, *J. Chem. Phy.* **1993**, *98*, 1372-1377; (b) A. D. Becke, *J. Chem. Phy.* **1993**, *98*, 5648-5652; (c) C. Lee, W. Yang, R. G. Parr, *Phys. Rev. B* **1988**, *37*, 785-789; (d) S. Grimme, J. Antony, S. Ehrlich, H. Krieg, *J. Chem. Phy.* **2010**, *132*, 154104; (e) S. Grimme, S. Ehrlich, L. Goerigk, *J. Comput. Chem.* **2011**, *32*, 1456-1465.

[8] F. Weigend, R. Ahlrichs, *Phys. Chem. Chem. Phys.* **2005**, *7*, 3297-3305.

[9] M. J. Frisch, G. W. Trucks, H. B. Schlegel, G. E. Scuseria, M. A. Robb, J. R. Cheeseman, G. Scalmani, V. Barone, G. A. Petersson, H. Nakatsuji, X. Li, M. Caricato, A. V. Marenich, J. Bloino, B. G. Janesko, R. Gomperts, B. Mennucci, H. P. Hratchian, J. V. Ortiz, A. F. Izmaylov, J. L. Sonnenberg, Williams, F. Ding, F. Lipparini, F. Egidi, J. Goings, B. Peng, A. Petrone, T. Henderson, D. Ranasinghe, V. G. Zakrzewski, J. Gao, N. Rega, G. Zheng, W. Liang, M. Hada, M. Ehara, K. Toyota, R. Fukuda, J. Hasegawa, M. Ishida, T. Nakajima, Y. Honda, O. Kitao, H. Nakai, T. Vreven, K. Throssell, J. A. Montgomery Jr., J. E. Peralta, F. Ogliaro, M. J. Bearpark, J. J. Heyd, E. N. Brothers, K. N. Kudin, V. N. Staroverov, T. A. Keith, R. Kobayashi, J. Normand, K. Raghavachari, A. P. Rendell, J. C. Burant, S. S. Iyengar, J. Tomasi, M. Cossi, J. M. Millam, M. Klene, C. Adamo, R. Cammi, J. W. Ochterski, R. L. Martin, K. Morokuma, O. Farkas, J. B. Foresman, D. J. Fox, Wallingford, CT, **2016**.

[10] J.-D. Chai, M. Head-Gordon, *Phys. Chem. Chem. Phys.* **2008**, *10*, 6615-6620.

[11] (a) J. Tomasi, B. Mennucci, R. Cammi, *Chem. Rev.* **2005**, *105*, 2999-3094; (b) A. V. Marenich, C. J. Cramer, D. G. Truhlar, *J. Phys. Chem. B* **2009**, *113*, 6378-6396.

[12] a) F Neese, WIREs Comput. Mol. Sci. 2012, 2, 73–78.; b) V. Barone, in Recent Advances in Density Functional Methods, Part I, Ed. D. P. Chong (World Scientific Publ. Co., Singapore, 1996)..

[13] (a) S. Stoll, A. Schweiger, *J. Magn. Reson.* **2006**, *178*, 42-55; (b) S. Stoll, R. D. Britt, *Phys. Chem. Chem. Phys.* **2009**, *11*, 6614-6625; (c) C. M. Marchand, U. Pidun, G. Frenking, H. Grützmacher, *J. Am. Chem. Soc.* **1997**, *119*, 11078-11085.

[14] G. te Velde, F. M. Bickelhaupt, E. J. Baerends, C. Fonseca Guerra, S. J. A. van Gisbergen, J. G. Snijders, T. Ziegler, *Journal of Computational Chemistry* **2001**, *22*, 931-967.

[15] (a) L. Zhao, M. Hermann, N. Holzmann, G. Frenking, *Coord. Chem. Rev.* **2017**, *344*, 163-204; (b) G. Frenking, M. Hermann, D. M. Andrada, N. Holzmann, *Chem. Soc. Rev.* **2016**, *45*, 1129-1144; (c) L. Zhao, S. Pan, N. Holzmann, P. Schwerdtfeger, G. Frenking, *Chem. Rev.* **2019**; (d) G. Frenking, F. M. Bickelhaupt, in *The Chemical Bond: Fundamental Aspects of Chemical Bonding* (Eds.: G. Frenking, S. Shaik), Eds. Wiley-VCH: Weinheim, **2014**, pp. 121-157; (e) L. Zhao, S. Pan, N. Holzmann, P. Schwerdtfeger, G. Frenking, *Chem. Rev.* **2019**, *119*, 8781−8845.
